# Public health competencies: what does the next generation of professionals deem important?

**DOI:** 10.1093/eurpub/ckae201

**Published:** 2025-03-25

**Authors:** Monica-Georgiana Brînzac, Marieke Verschuuren, Lore Leighton, Robert Otok

**Affiliations:** EUPHA, Utrecht, Netherlands; Department of Public Health, Babeș-Bolyai University, Cluj-Napoca, Romania; EUPHA, Utrecht, Netherlands; ASPHER, Brussels, Belgium; ASPHER, Brussels, Belgium

## Abstract

To adequately address many complex societal challenges, the public health workforce needs to learn new competencies. It is of particular interest to know what young people consider important in this regard, as they represent the future generation of public health professionals. Public health students and early career professionals in Europe were asked by means of an online questionnaire which of the competencies included in the 2020 WHO-ASPHER Competency Framework for the Public Health Workforce in the European Region they deemed most important for their future careers, and how well prepared they felt to execute these competencies in practice. In total, 127 respondents were included from 25 countries. They ranked *Promoting health*, *Science and practice*, and *Leadership and systems thinking* as the most important competency areas for their future careers. The first two were also the competencies for which the respondents felt best prepared, while they felt less prepared for *Leadership and systems thinking*. Other competencies that they felt less prepared for were *Law, policies, and ethics*; *Governance and resource management*; and *One health and health security*. This study shows a mismatch between what young professionals deem most important and what they feel best prepared for. The respondents did not feel well prepared for several competencies that are vital for a well-equipped future public health workforce. Public health in Europe would benefit from a unified public health curriculum that implies a list of mandatory competencies and a closer collaboration between academia with students and young professionals and the public health sector.

## Introduction 

Even before the COVID-19 pandemic, our world was transforming rapidly and profoundly. Changing demographics, globalization, technological developments, climate change, conflicts, increasing migration, shifting public opinions on the value of scientific evidence, influence of social media, a political arena that increasingly seems to focus on highlighting differences rather than looking for common ground: all these trends result in massive and complex societal challenges, which place great strain on our often underfunded and understaffed public health and health care services [1, 2]. In addition, the Pan-European Commission on Health and Sustainable Development, known as the Monti Commission, reviewed the evidence on the impact of the pandemic to formulate lessons learned and a call to action. They concluded that the COVID-19 crisis has reminded us of the vulnerability of societies, economies, and health systems, the weaknesses of our current systems of governance at national and global levels, and shone a light on the deep fault lines that exist in many societies [3]. The *Lancet Commission on lessons for the future from the COVID-19 pandemic* took an even more critical stance, finding ‘Widespread failures during the COVID-19 pandemic at multiple levels worldwide have led to millions of preventable deaths and a reversal in progress towards sustainable development for many countries’ [4, 5].

One issue that has been clearly highlighted by the pandemic is that a trained, motivated, and equipped public health workforce is essential [3]. Among the Lancet Commission recommendations is a call for ‘strong health education for health promotion, disease prevention, and emergency preparedness’ [4], while a recent European Centre for Disease Prevention and Control (ECDC) report makes the case for investment in the public health workforce with improvements and investment in preparedness planning and the need for a formalized decision-making and crisis management structure that supports intersectoral work, as well as increasing the capacities in risk communication and community engagement [6]. This illustrates that the workforce does not only have to be prepared for infectious disease outbreaks, but for all the societal changes that impact on population health as described above.

**Figure 1.**
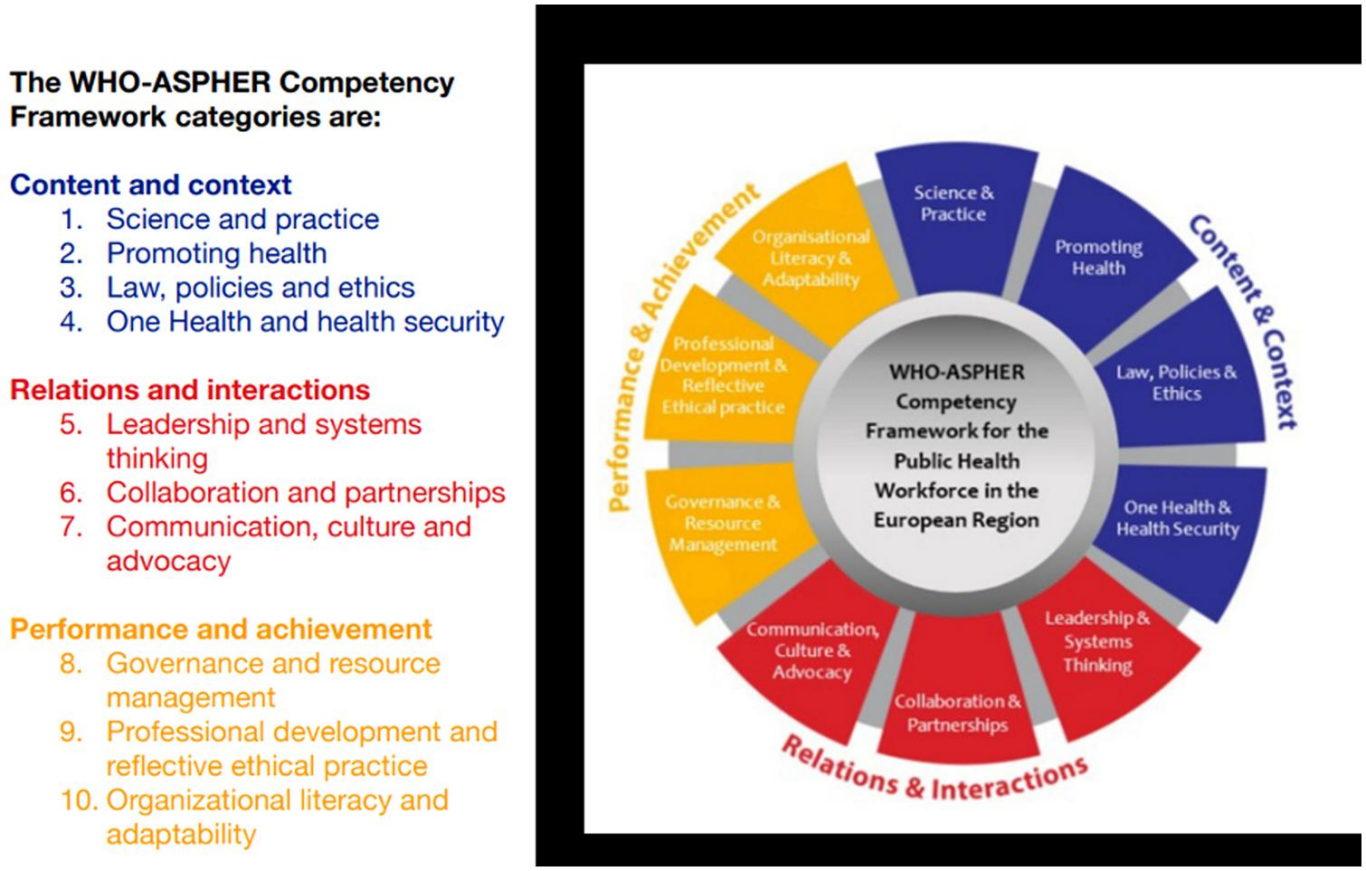
Categories, sections, and competencies of the WHO-ASPHER Competency Framework. Source: World Health Organization Regional Office for Europe [14].

To concretize what this means for the public health workforce specifically, various competency frameworks for public health professionals have been developed and/or updated in recent years [7–11]. Competencies represent combinations of individual attributes (such as knowledge, skills, and personal or professional attitudes) individuals require to undertake the professional role they are expected to fulfil [12, 13]. A key European reference among these frameworks is the ‘WHO-ASPHER Competency Framework for the Public Health Workforce in the European Region’, which was published in 2020 [14] and incorporated into the WHO-ASPHER Roadmap to Professionalizing the Public Health Workforce in the European Region as one of the pillars to professionalize the public health workforce [15]. This comprehensive framework focuses on three major categories, which are then further subdivided into 10 sections, which contain a total of 84 competencies (see Fig. 1). Elements such as *One health*, *Leadership and systems thinking*, *Collaboration and partnerships*, and governance illustrate that the required competences for the public health workforce of today and tomorrow have expanded greatly from the traditional infectious disease control and health promotion focus.

These competencies should be taken into account by higher education institutions for public health education, training, and capacity building; by public health institutes and others responsible for policy making, implementation, and public health workforce planning; by human resources and employers in professional and practice settings; and by professional representation bodies.

For the development of apt and appropriate curricula and programmes, it is important to learn what the upcoming generation of early career professionals think is most relevant and what they feel they need to be well prepared for their future careers and advancement. To ensure that the next generation of public health professionals is ready for the complex tasks they will be confronted with, early career professionals should be genuinely involved [16]. Therefore, this study was conducted in which public health students and early career professionals were asked which of the competencies in the WHO-ASPHER Framework they deem to be most important.

## Methods

The study had a cross-sectional design, using quantitative methods, through an online survey, available in the Supplementary Material. The survey was developed collaboratively by the European Public Health Association (EUPHA) and the Association of Schools of Public Health in the European Region (ASPHER) and underwent several rounds of feedback by three EUPHA and ASPHER staff members other than the developer of the questionnaire, including one young professional representing the target audience. The final form of the structured questionnaire included 10 questions divided into two sections. The first section with three questions requested demographic information. The second section of seven questions focused on the WHO-ASPHER Competency Framework for the Public Health Workforce in the European Region. The questionnaire was developed in English only, while guaranteeing that all questions safeguard the rights and welfare of the respondents.

The target population of the study were students and early career professionals in public health. The study relied on a convenience sample with the sample size driven by time. The inclusion criteria were (1) to be a student in public health or an early career public health professional with 5 years or less experience; (2) to have completed studies in one of the WHO European Region countries.

The survey was disseminated online among EUPHA, EUPHAnxt (EUPHA’s network for students and young professionals), and ASPHER networks via Twitter, Instagram, Facebook, LinkedIn, ASPHER mailing lists, and EUPHA and EUPHAnxt newsletters, ensuring that it includes and reaches as many students, young professionals, and early career professionals as possible. Participation in the survey was voluntary and anonymous.

Data was collected using Microsoft Forms from 25 May 2023 until 29 June 2023. No time constraints were enforced for the completion of the survey, and the survey could be re-accessed without loss of already filled-in data to facilitate its finalization. The results were available only to the research team in a password-protected environment.

Descriptive statistics were conducted to report the results. Percentages and frequency distribution were used to characterize nominal and ordinal data. All analyses were performed in Microsoft Excel.

## Results

Out of the total of 157 respondents, 127 were included, meaning that 80.89% of respondents met the inclusion criteria described above. The included respondents came from 25 different countries.

Table 1 depicts the characteristics of the sample in terms of the country in which the included respondents conducted the majority of their studies and the country in which they currently reside. It is noticeable that a share of the respondents moved to a different country after completion of studies, but only for 10.23% of the sample. Of the 13 cases of relocation, four moved outside the WHO European region—Canada, the United Arab Emirates, and Nigeria.

**Table 1.** Countries in which the included respondents conducted their studies and their current residence country

Country	No. of studies	No. of residence
1. Austria	3	3
2. Belgium	2	3
3. Cyprus	1	0
4. Czechia	1	1
5. Finland	1	1
6. Germany	26	24
7. Greece	1	1
8. Hungary	1	1
9 Ireland	5	5
10. Italy	31	28
11. Lithuania	3	3
12. Netherlands	4	3
13. North Macedonia	1	1
14. Poland	1	1
15. Portugal	6	6
16. Republic of Moldova	1	0
17. Romania	10	11
18. Russian Federation	1	0
19. Serbia	3	3
20. Slovakia	2	2
21. Spain	1	1
22. Sweden	1	3
23. Switzerland	11	14
24. Türkiye	3	3
25. United Kingdom of Great Britain and Northern Ireland	7	4

**Figure 2.**
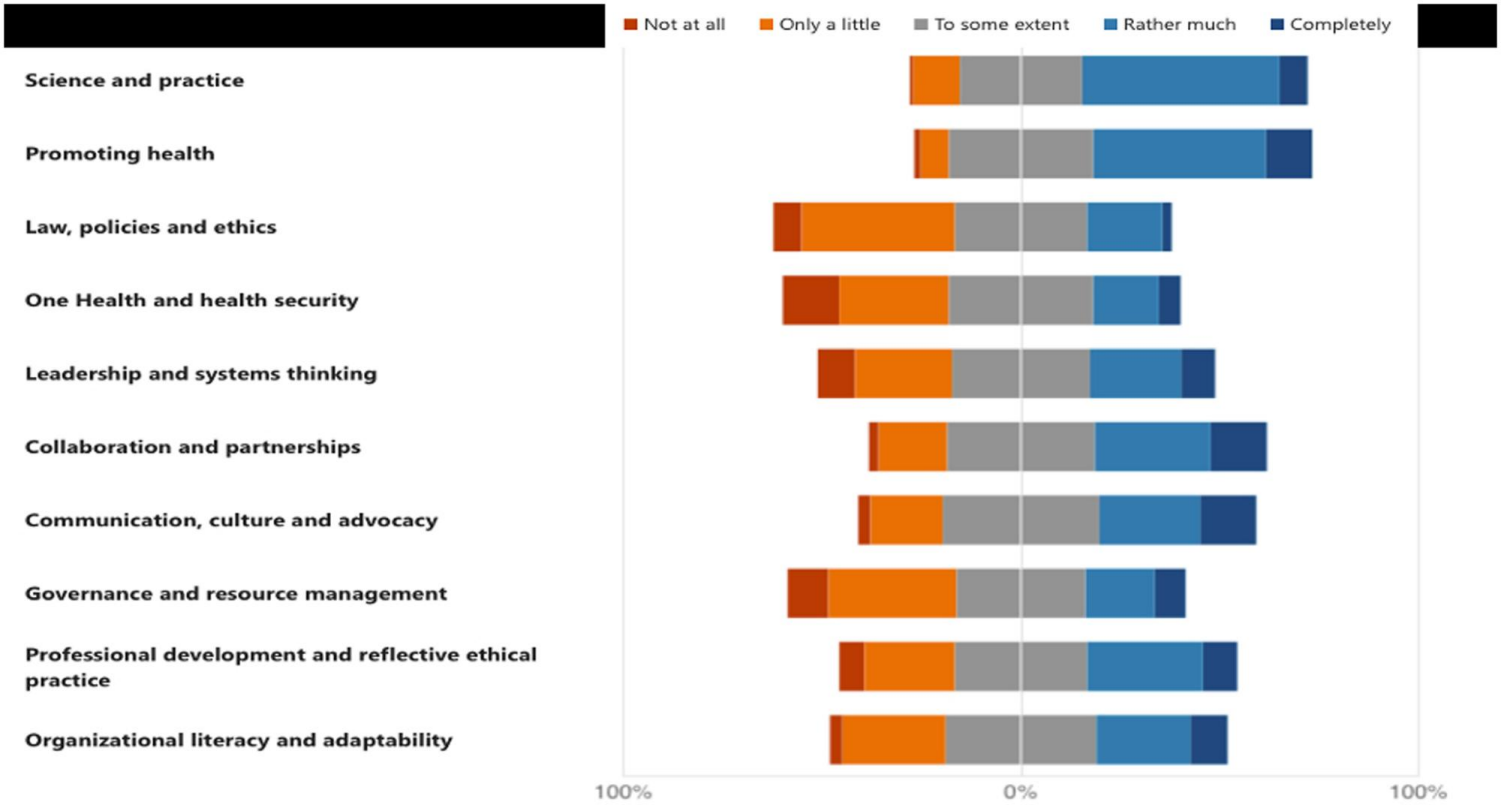
Self-assessed level of the WHO-ASPHER competencies.

Figure 2 illustrates the level at which the respondents feel they are prepared to use each of the competency areas in their current and/or future professional life. The *science and practice* competency, defined in the framework as ‘epidemiology of communicable and noncommunicable diseases; demography; biostatistics; qualitative and quantitative research methods; assessment, analysis and evaluation; evidence-based research; measurement, monitoring and reporting; health indicators; health systems; population health; health inequalities’ [14], is the highest-ranking dimension of the 10, with half of the sample (56.7%) assessing themselves as rather much (49.6%) or completely prepared (7.1%). Following closely second was the *promoting health* competency, ‘Education and promotion through social participation; health literacy at the community, organization and individual levels; citizen empowerment; health needs assessment; screening and secondary prevention; evaluation of health promotion interventions and programmes’ [14], with (55.1%) of the sample assessing themselves as rather much (43.3%) or completely prepared (11.8%). Scoring equally, *collaboration and partnerships*, ‘effective collaboration; building alliances and partnerships; networking and connecting; working with and building interdisciplinary and intersectoral networks; dealing with and managing stakeholders’ [14] and *professional development and reflective ethical practice*, ‘professional and reflective practice; continuing professional development; life-long learning; values; ethical professional conduct’ [14], were self-assessed as the third competencies the students and early career professionals feel most prepared to use (29.1%). On the other hand, the sample ranks their *law, policies, and ethics*; *governance and resource management*; and *one health and health security* competencies as the least developed and prepared to use in professional circumstances.

The knowledge behind the competencies was reported to be predominantly obtained from *professional experiences* for a third of the sample (33%), while *classroom instruction* is the second most important method through which respondents have learned about each competency (26%). *Internships and fellowships* were the third most common source of knowledge regarding competencies (22%). Respondents were able to select multiple options here.

**Figure 3.**
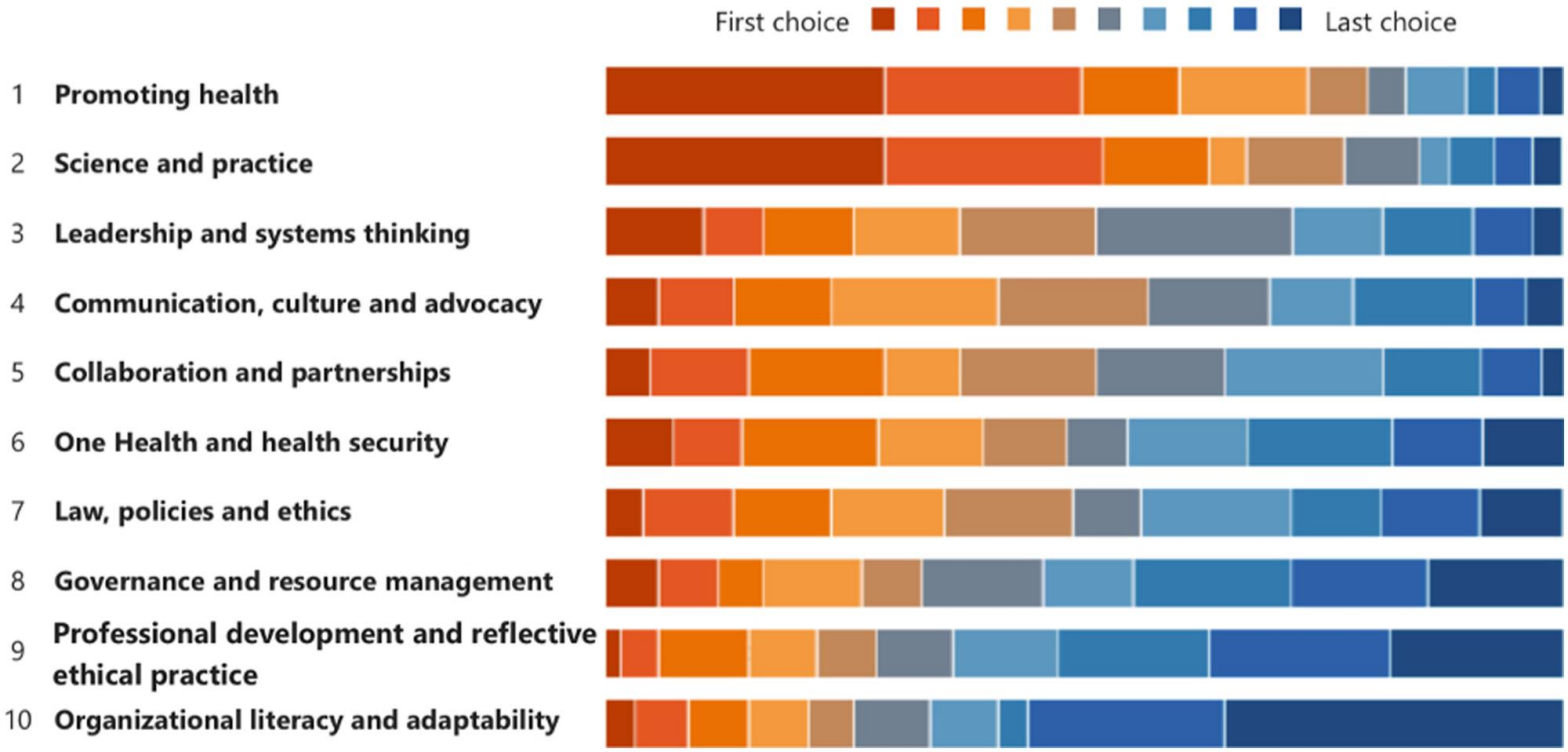
The most important WHO-ASPHER competencies in professional life.

Figure 3 shows which of the competencies of the WHO-ASPHER Framework the respondents think are most important for their professional life. Here, we see some overlap with the competencies for which the respondents feel most prepared. Similar to their self-assessment, promoting health and science and practice are closely ranked, with very small differences in terms of their ranks. *Leadership and systems thinking*, ‘vision, mission and strategy; individual task-team work; leading change and innovation; understanding and applying the theories of complex systems in practice; organizational learning and development; people development; emotional intelligence’, was ranked as the third most important competency.

In response to the question, ‘What would make you feel better prepared for your current/future public health professional life?’ *Professional experience/exposure during studies* was mentioned most often by almost one-third (29%) of the respondents. *Mentorship* was identified by 23% of the respondents as the second most important initiative that would be highly beneficial to their preparedness. The third most important need for the students/early career professionals to feel better prepared was *networking opportunities* (18%). The fourth most important means was young professional networks (15%), and following closely as the fifth was conference/meeting attendance (14%).

When asked if the WHO-ASPHER Competency Framework for the Public Health Workforce in the European Region adequately represents the skills needed in public health practice, the majority (58%) of the sample agrees that the framework adequately represents the needed skills, while a third (32%) do not know and 9% disagree. One respondent stated that the framework is representative to some extent.

Omissions identified by the respondents in the framework include emotional intelligence, organizational citizenship, behaviour, research, clinical work, patient and community engagement, healthcare management, global health, language skills, professional experiences while studying, job hunt skills, interview management, human resources, negotiation skills, knowledge of the labour market, opportunities to practice, psychology and coaching, public health advocacy, and social and behavioural sciences. Additional suggestions made include a unified public health curriculum that implies a list of mandatory competencies and a closer collaboration between academia and industry/the public sector.

## Discussion

The respondents included in this study ranked the competencies *Promoting health*; *Science and practice*; and *Leadership and systems thinking* as the most important for their future careers. Promoting health and Science and practice were also the two competencies for which the respondents felt best prepared, while they felt less prepared for *Leadership and systems thinking* which was ranked as the third most important competency. This latter finding shows a mismatch between what young professionals deem most important and what they feel best prepared for. Other competencies that they felt less prepared for were *Law, policies, and ethics*; *Governance and resource management*; and *One health and health security*. Yet, these competencies are vital for a well-equipped public health workforce in the future.

These results are striking in light of similar findings from a 2011 study of ASPHER and EUPHA members on demand for public health courses for life-long learning, which found a gap in supply and demand of courses for *Management, planning, organization*; *Leadership*; *Analysis and critical thinking*, among others [17]. The continued gap found in *Leadership and system thinking* as well as in *Governance and resource management* indicates a sustained and unfortunate lack of attention to competencies most needed and sought out by public health professionals. Meanwhile, differences in the demand for courses in the areas of Law and ethics; and Environmental and occupational health, which were not found to have a supply and demand gap in 2011 as compared to the finding in this study of a gap in competencies for *Laws, policies and ethics*; and *One health and health security* indicate the need for public health training and education to react nimbly in order to respond to rapidly changing demands on the public health workforce.

Therefore, the results of this study underline the importance of including students and early career professionals in curriculum development and decision-making processes, which has recently been stressed by other authors [18, 19], and are in line with a wider acknowledgement of the need to involve young people in decision-making processes regarding health. The global WHO Youth Council and Youth4Health, the WHO Regional Office for Europe’s youth initiative, are illustrative examples of this movement. This study also underscores the need to routinely revise and review competencies required by the public health workforce in a changing world. ASPHER is actively involved in this task through ASPHER’s Europe Core Competencies List for the Public Health Professional, which has undergone several revisions [20] as well as ASPHER’s Core Curriculum Project (CCP). CCP is currently underway to design a unified Public Health Core Curriculum based on European values in collaboration with colleagues at University College Dublin with partner organizations and in consultation with ASPHER’s network of schools of public health and a wide range of public health experts and public health professionals (including recognized networks of young professionals in Europe such as ASPHER Young Professionals, EUPHAnxt, Young Forum Gastein, and EuroNetMRPH). The results of this effort will be launched during the 2024 European Public Health Conference in Lisbon and will help to guide ASPHER member schools and programmes of public health to improve and update their curriculum offer to students and for continuing education and training.

In its Statement on the Erosion of Public Health Systems, ASPHER noted that the discipline of public health lies ‘at the crossroads of science and policy, it offers evidence-based information for better decision making in the matters of health … continuous exchanges between decision makers and public health experts are always required to improve and protect the health of the public’ [21]. This is verified by the responses of early career professionals, who identify the importance of leadership; law policies and ethics; and governance and resource management as key competencies to carry out public health work. However, the fact that they feel unprepared in these areas, as well as in the domain of *One health and health security*, must be a call for attention to education and training in these competency areas, both in regular degree programmes and in continuing education for public health professionals, so that they are empowered and capable to effectively impact on and be responsive to current and future health threats. If we do not adequately address the competencies needed for public health and invest in its functioning as a profession, then we cannot expect to adequately confront threats to the public’s health.

The respondents of this study indicated that professional experience/exposure during studies, mentorships, and networking opportunities are important means to improve their competencies and be better prepared for their future careers. The importance of mentorship structures was also mentioned by Wong *et al.*, who identified a lack of mentorship, inaccessibility, tokenisation, and a non-representative workforce as the main barriers for incorporating young professionals into the public health workforce [16]. Mentorship relations as well as increased networking and training opportunities are also seen as crucial by the World Federation of Public Health Associations in their recommendations for meaningful engagement of young professionals [22].

EUPHA, together with its network for students and early career professionals, EUPHAnxt, recently launched an online mentoring programme. More than 160 students and early career professionals applied for the programme, illustrating the great need for such initiatives. EUPHA and EUPHAnxt also run an internship and fellowship programme and provide several networking opportunities for students and young professionals, such as during the annual European Public Health Conferences.

Response to the needs of young professionals as demonstrated in this paper is a critical part of ASPHER’s mission and has been enshrined in the 2025 strategy, which sets the Next Generation—Students & Alumni as one of three key enabling areas. Under the strategy, ASPHER has initiated a Young Professionals Programme (YPP). Participants are paired with ASPHER faculty mentors and take part in ASPHER task forces and working groups. A YPP member has been co-opted into the ASPHER Executive Board, and likewise, the EUPHAnxt coordinator is a member of the EUPHA Executive Council as well as the European Public Health Conference Executive Board. ASPHER interns and fellows contribute directly to development of signature ASPHER-led reference materials on competencies and core curriculum. Moreover, the Association, in collaboration with EUPHA, is continuing the long-standing ASPHER Young Researchers Forum (begun in 2009), which provides a platform for young researchers to present their work at the European Public Health Conference and other member events, as well as the ASSETS Mentoring Summer School (begun in 2019), which provides a unique intensive mentoring experience with networking across other Brussels-based organizations for motivated early career professionals.

The outcomes of this study underline the importance of continuous investment in and further development of these activities to match the needs of the next generation of public health professionals. The WHO-ASPHER Roadmap to Professionalizing the Public Health Workforce provides a pragmatic guide to build capacity and develop the public health profession—we call on all those concerned with the health of the population and the planet (governments, national and local authorities, international bodies, associations, and schools of public health) to join us to ensure that public health professionals are adequately trained, empowered, and resourced to lead us to a healthier future.

## Limitations

It must be noted that the results of this study cannot be regarded as representative of the entire group of public health students and early career professionals in Europe. For this study, the authors wanted to obtain quick insights into the perceptions of public health students and early career professionals. Therefore, an online survey was applied, and a convenience sample that was recruited from and through the EUPHA and ASPHER networks was used. This approach allowed for easy recruitment of respondents and rapid data collection, which resulted in a fair and informative number of responses in little more than 1-month time. However, the chosen methodology limits the generalizability of findings. In addition, the respondents mainly came from the Western part of the WHO European Region, meaning that the opinions of students and early career professionals from the Eastern part are not reflected in the results presented. The fact that the questionnaire has been made available in English only may have contributed to the lack of response from Eastern Europe. The authors aimed to keep the questionnaire concise so as not to discourage respondents from participating. However, this means that the questionnaire is by no means exhaustive and only addresses a limited number of questions related to the respondents’ views on and experiences with competencies. This means that the results of this study should be seen as indicative and as the starting point for further discussions with students and early career professionals about how public health competencies and related curricula and trainings can be amended in such a way that they truly meet the needs of young professionals.

## Supplementary Material

ckae201_Supplementary_Data

## Data Availability

The data that support the findings of this study are available from the first author, MGB, upon reasonable request.
