# Supplementary material for: Public health competencies: what does the next generation of professionals deem important?
Source: Eur J Public Health. 2025 Mar 25;35(Suppl 2):ii11–6. doi: 10.1093/eurpub/ckae201 (PMC11933798; doi:10.1093/eurpub/ckae201)
Supplement: ckae201_Supplementary_Data [file ckae201_supplementary_data.docx]

**Supplementary materials**

**Demographics**

1. In which country did you conduct the majority of your studies?

Albania

Andorra

Armenia

Austria

Azerbaijan

Belarus

Belgium

Bosnia and Herzegovina

Bulgaria

Croatia

Cyprus

Czechia

Denmark

Estonia

France

Finland

Georgia

Germany

Greece

Hungary

Iceland

Ireland

Israel

Italy

Kazakhstan

Kyrgyzstan

Latvia

Lithuania

Luxembourg

Malta

Monaco

Montenegro

Netherlands

North Macedonia

Norway

Poland

Portugal

Republic of Moldova

Romania

Russian Federation

San Marino

Serbia

Slovakia

Slovenia

Spain

Sweden

Switzerland

Tajikistan

Türkiye

Turkmenistan

Ukraine

United Kingdom of Great Britain and Northern Ireland

Uzbekistan

Other (fill in)

1. In which country do you currently reside?

Albania

Andorra

Armenia

Austria

Azerbaijan

Belarus

Belgium

Bosnia and Herzegovina

Bulgaria

Croatia

Cyprus

Czechia

Denmark

Estonia

France

Finland

Georgia

Germany

Greece

Hungary

Iceland

Ireland

Israel

Italy

Kazakhstan

Kyrgyzstan

Latvia

Lithuania

Luxembourg

Malta

Monaco

Montenegro

Netherlands

North Macedonia

Norway

Poland

Portugal

Republic of Moldova

Romania

Russian Federation

San Marino

Serbia

Slovakia

Slovenia

Spain

Sweden

Switzerland

Tajikistan

Türkiye

Turkmenistan

Ukraine

United Kingdom of Great Britain and Northern Ireland

Uzbekistan

Other (fill in)

1. Are you a current student in public health or an early career professional (5 years or less experience)?

Yes

No (skip to the end of the questionnaire)

**WHO-ASPHER competency framework**

1. Please select to which level you feel you are prepared to use each of the competencies in your current and/or future professional life

|  | Not at all | Only a little | To some extent | Rather much | Completely |
| --- | --- | --- | --- | --- | --- |
| Science and practice |  |  |  |  |  |
| Promoting health |  |  |  |  |  |
| Law, policies and ethics |  |  |  |  |  |
| One Health and health security |  |  |  |  |  |
| Leadership and systems thinking |  |  |  |  |  |
| Collaboration and partnerships |  |  |  |  |  |
| Communication, culture and advocacy |  |  |  |  |  |
| Governance and resource management |  |  |  |  |  |
| Professional development and reflective ethical practice |  |  |  |  |  |
| Organizational literacy and adaptability |  |  |  |  |  |

1. Of the 10 competencies, which do you feel are the most important to your current and/or future professional life? (rank in order of importance)

Science and practice

Promoting health

Law, policies and ethics

One Health and health security

Leadership and systems thinking

Collaboration and partnerships

Communication, culture and advocacy

Governance and resource management

Professional development and reflective ethical practice

Organizational literacy and adaptability

1. Via which methods do you feel you learned about each competency?

Classroom instruction

Internship, fellowship

Professional experience

Networks, societies

Other (fill in)

1. Do you feel as though the competency framework adequately represents the skills you need in your public health practice?

Yes

No

I don’t know

Other (fill in)

1. If no, please share any missing elements

_________________________________

1. What would make you feel better prepared for your current/future public health professional life?

Professional experience/exposure during studies

Young professional networks

Access to mentorship

Conference/meeting attendance

Networking opportunities

Other (fill in)

1. Is there anything else you’d like to share?

_________________________________
